# Dynamic Changes in Soil Phosphorus Accumulation and Bioavailability in Phosphorus-Contaminated Protected Fields

**DOI:** 10.3390/ijerph191912262

**Published:** 2022-09-27

**Authors:** Hongyue Liang, Chen Wang, Xinrui Lu, Chunmei Sai, Yunjiang Liang

**Affiliations:** 1 College of Agriculture, Yanbian University, Yanji 133002, China; 2 Northeast Institute of Geography and Agroecology, Chinese Academy of Sciences, Changchun 130012, China; 3 College of Pharmacy, Jining Medical University, Rizhao 276826, China

**Keywords:** greenhouse, phosphorus pollution, accumulated phosphorus, inorganic phosphorus, spinach, the threshold

## Abstract

Soil phosphorus accumulation resulting in a high risk of phosphorus pollution is due to high multiple vegetable cropping indexes and excessive fertilizer input in protected fields. Therefore, this study explored the bioavailability of soil-accumulated phosphorus to improve fertilization and reduce the risk of soil phosphorus contamination in protected fields. A field trial was performed in Yanbian Prefecture, China to study the phosphorus bioavailability after continuous spinach planting without phosphate fertilizer applications. Results indicated that with increasing numbers of planting stubbles, soil inorganic phosphorus and occluded phosphorus changed little, while water-soluble and loose phosphorus, aluminum-phosphate, iron-phosphate, and calcium-phosphorus decreased first and then increased. Soil available phosphorus declined linearly. For planting spinach in protected fields, the threshold of soil phosphorus deficiency is 200 mg kg^−1^. A soil phosphorus supply potential model was established between *x* (the soil available phosphorus) and *y* (the numbers of planting stubbles): *y* = 6.759 + 0.027*x*, R = 0.99, which can be used to predict how planting stubbles are needed to raise the soil available phosphorus above the critical value of phosphorus deficiency for spinach. These results will provide the theoretical guidance for rational phosphorus fertilizer applications and control agricultural, non-point pollution sources in protected fields.

## 1. Introduction

Vegetable production in protected fields is an efficient and intensive production model. The area of vegetable production in protected fields has increased fivefold over the last ten years due to the ability to control climatic conditions in a closed environment and thus increase food production [1]. In addition, as a large agricultural country, China’s protected fields have increased to 20% of the total vegetable production area in China over the last 20 years [2]. High temperatures, high evaporation, and relatively closed environments have long been associated with protected fields. In these environments, soil phosphorus accumulation increases in proportion to planting years [3], resulting in a high risk of phosphorus pollution due to the high multiple cropping index and excessive chemical fertilizer input.

Phosphorus is required for plant growth and development. Soil provides most of the phosphorus required by plants, but to maintain high yields, vegetable farmers continue to apply phosphorus fertilizers to the soil [4]. A soil survey discovered that the Olsen-P content in the 0–20 cm soil layer of greenhouse vegetable growing fields in Beijing suburbs was as high as 358 mg kg^−1^ [5], while the critical Olsen-P value of the soil in Chinese protected vegetable fields was in the range of 46.0–58.0 mg kg^−1^ [6]. Excessive phosphorus fertilizer input, which exceeds the needs of plants and soils, not only fails to achieve the goal of maintaining increased production [7], but also causes a large accumulation of phosphorus in the soil [8] and increases the potential for phosphorus loss [9]. In addition, excessive phosphorus fertilizer input will lead to phosphorus accumulation; the utilization rate of phosphate fertilizer is typically less than 20% [10]. Large amounts of phosphorus accumulate in water bodies, primarily through irrigation or rainfall leaching loss [11], resulting in agricultural phosphorus pollution [12,13]. Phosphorus accumulation is currently a common phenomenon in the soils of protected fields. As a result, it is critical to control phosphorus inputs to the soil and reduce phosphorus leaching from the soil.

Many scholars have prioritized continuous, long-term field research as their primary research investment, and their findings have provided a theoretical foundation for sustainable cropping patterns, accumulated soil phosphorus removal, and soil fertility issues. There is symmetry between the depletion and accumulation rates of plant available phosphorus in soils that do not receive phosphorus fertilization [14]. Soil contains two types of phosphorus: inorganic phosphorus and organic phosphorus, both of which can be used as a source of soluble phosphorus. The use of ^31^P tracer atoms to investigate the form and availability of phosphorus in calcareous soil has revealed that after soluble phosphorus enters the soil, its availability decreases with increasing planting stubble, and it is rapidly converted into inorganic phosphorus, the main form of which is Ca-P, Al-P, and Fe-P type phosphates [15]. According to recent research, the transformation and transfer of phosphorus in soil are primarily influenced by soil pH and soil organic matter (SOM) content [16]. Nowadays, academics favor using continuous cropping without fertilization for field research. Srinivasarao et al. [17] studied an area over 20 years of continuous cropping without K fertilizer under arid conditions and discovered that soil available K reduced by half. In addition, a trial was conducted to study the effects of intercropping on maize and fata bean under continuous cropping without P fertilizer. After four years, in soil P pools, the amounts of both inorganic and organic P decreased a lot. Maize and fata bean intercropping without P fertilizer system increased P utilization compared with cropping alone [18]. Experiments have shown that continuous cropping without the use of phosphate fertilizer can redistribute phosphorus in the topsoil and plants, effectively reduce phosphorus leaching in the short term [19,20], and thus reduce the risk of phosphorus pollution.

Developing a model for predicting soil available phosphorus and phosphorus deficiency, as well as determining the critical phosphorus concentration, can provide a theoretical foundation for effectively guiding rational fertilization in protected agriculture. There are numerous methods for evaluating soil phosphorus fertilizers, but none of them can quantify the phosphorus requirement of plants. Ejraei et al. [21] proposed an innovative “Integrated Plant-Soil System” (IPSS) that quantitatively calculates the phosphorus required by plants based on the correlation between soil phosphorus, soil properties, and phosphorus in plant organs and which is suitable for both high-yielding and phosphorus-deficient orchards. Ma et al. [22] modeled a variation of soil Olsen-P over multi-year periods including years with and without phosphate fertilizer applications using long-term experimental data of wheat-maize rotation systems under five different climatic conditions in China. The concentration decreased to 3 mg kg^−1^ as the cultivation time increased and remained constant. The phosphorus nutritional index (PNI) was developed as a plant-based index derived from the critical phosphorus concentration (PC) model to predict the feedback effect of corn on phosphorus fertilizer. Following farm verification, the critical value of corn phosphorus concentration was determined to be 0.9, below which corn responds positively to phosphorus fertilizer. [23]. The PC model was also used to calculate the available phosphorus in the soil tillage layer and to draw the critical phosphorus curve. The soil- and crop-based evaluation methods were evaluated concurrently, demonstrating that process-based evaluation was more relevant and reliable than chemical extraction [24].

In recent years, a large area of dark-brown soils in the Yanbian area have been converted into protected fields, which, characterized by phosphorus accumulation, face the risk of rising phosphorus pollution. However, not much is known about the threshold of soil phosphorus deficiency in these soils, and a soil phosphorus supply potential prediction model based on the known soil Bray-P content has not been developed. Therefore, this paper studies the variation of soil inorganic phosphorus, soil available phosphorus, and phosphorus bioavailability after continuous spinach planting with no phosphate fertilizer application in protected fields on dark-brown soils to characterize the bioavailability of accumulated phosphorus. The outcomes could give theoretical guidance and reference for rational phosphorus fertilizer application and enrichment of the soil phosphorus abundance index system in these protected agricultures.

## 2. Materials and Methods

### 2.1. Experimental Soils

1. Soils for research on the characteristics of accumulated phosphorus: typical plastic greenhouses cultivated on dark brown soil in Longjing City, Yanbian Prefecture, Jilin Province were chosen, and at each, soil samples were collected at depths of 0–20 cm and 20–40 cm. Three protected field soil samples were collected from Longchi village (No. 1, 2, and 3), four from Longhai village (No. 4, 5, 7, and 8), and one from Longfeng village (No. 6). Simultaneously, a farmland (i.e., non-protected, open-air vegetable field) soil sample was collected from Longhai village as a control (No. 9). For each sample, the soil total phosphorus, available phosphorus, different forms of inorganic phosphorus, and organic phosphorus were measured, and for the protected fields, the characteristics of the soil accumulated phosphorus were also analyzed.
2. Soils for the continuous spinach planting without phosphate fertilization experiment: Soil was collected from 17 greenhouses in Longjing City (7 in Longchi village and 10 in Longfeng village). These are protected-field soil samples with an obvious phosphorus accumulation gradient and similar texture. At the same time, there were three places in Longfeng village (No. A, B, and C) and two places in Longchi village (No. D and E). A farmland soil sample was used as a control (No. F). Table 1 lists the basic properties.

### 2.2. Experimental Crops

The test crop was a first-generation hybrid spinach (*Spinacia oleracea* L.) imported from Denmark, and the seeds were bought from the Changchun Institute of Modern Agricultural Science and Technology. Four planting stubbles were completed from May to December 2008, and two from March to November 2009.

### 2.3. Experimental Design and Methods

Protected-field soil samples representing five levels of accumulated phosphorus in the plow layer, along with a farmland soil sample as a control, were used in a continuous-planting pot experiment using spinach. The experiment was conducted at the Agricultural College of Yanbian University’s intelligent greenhouse. Six planting stubbles (planting, growth, and then harvesting) of spinach were completed in succession with no phosphate fertilizer applied, and a farmland control treatment was established at the same time. The specific methods were to lay a layer of small gravel on the bottom of a basin, resulting in a combined weight of 700 g for each basin. Air-dried soil from each sample was placed in pots until 42 pots representing each soil treatment were filled, 3.5 kg for each pot. Water and non-phosphorus-containing fertilizer were applied together: the calculated amount of fertilizer was dissolved into the water before application. We planted 9 spinach plants evenly in each pot, which were then placed in sunny positions in the greenhouse where the sunlight was as uniform as possible. Watering was done sparingly and frequently during the cultivation process to avoid fertilizer loss caused by leaching. In addition, water that accumulated at the basin’s bottom was collected for reuse. After each planting stubble, soil samples, and plants were collected, the remaining soil was used to continue the experiment.

### 2.4. Analysis of Soil Samples

Soil pH was analyzed by potentiometry. Organic matter content was analyzed by the potassium dichromate volumetric method. Available phosphorus was quantified by NH_4_F-HCl ex-traction phosphomolybdic blue colorimetry, and available potassium by NH_4_OAc extraction and flame spectrophotometry. The content of alkali-hydrolyzed nitrogen was measured using the alkali-hydrolyzed diffusion method. The total phosphorus content was determined by HClO_4_-H_2_SO_4_ spectrophotometry, total nitrogen content by HClO_4_-H_2_SO_4_ de-boiling and semi-microdenitrification distillation, and the total potassium content was analyzed using NaOH flame spectrophotometry. Lastly, inorganic phosphorus was analyzed by the improved Petersen method.

### 2.5. Data Analysis

SPSS 21.0 and Excel were used for classical statistical analyses. The model was formulated using Curve Expert 1.3 software. Origin 2021 was used for making the graph.

## 3. Results

### 3.1. Soil Accumulated Phosphorus Characteristics in Protected Fields

#### 3.1.1. Soil Phosphorus Composition in Protected Fields

Table 2 shows the total phosphorus, inorganic phosphorus, organic phosphorus, available phosphorus, and the proportion of total phosphorus in each studied area’s 0–20 cm soil layer.

The data in the table have low standard errors and are dependable; as a result, they can be analyzed in the following step. Total phosphorus content in the 0–20 cm layer of the protected-field soils was 0.80–2.49 g kg^−1^, which was significantly higher than that of the farmland soil. The inorganic phosphorus content in the soils of the protected fields was 0.63–1.76 g kg^−1^, accounting for 50.5–86.9% of the total phosphorus, whereas the inorganic phosphorus content in the farmland soil was only 0.27 g kg^−1^, accounting for 75.1% of the total phosphorus. This demonstrates that the protection provided by the greenhouses is beneficial. Inorganic phosphorus accumulation in the protected-field soils was also more serious than in open-air field soils. The soil organic phosphorus content in the protected field was 0.16–1.21 g kg^−1^, accounting for 13.1–49.5% of total phosphorus, whereas that of the farmland soil was only about 0.09 g kg^−1^, accounting for 24.9% of total phosphorus. The organic phosphorus content of the protected-field soils was clearly greater than that of the farmland soil. The available phosphorus content of the protected-field soils was 0.04–0.53 g kg^−1^, while the available phosphorus content of the farmland soil was around 0.03 g kg^−1^. The soil available phosphorus content was higher in the protected-field soils than in the farmland soil.

Table 3 shows the total phosphorus, inorganic phosphorus, organic phosphorus, available phosphorus, and the proportion of total phosphorus in each studied area’s 20–40 cm soil layer.

The data have been examined and are dependable. The total phosphorus content of the 20–40 cm layer of the protected-field soils was 0.6–2.02 g kg^−1^, whereas the total phosphorus content of the farmland soil was approximately 0.34 g kg^−1^. Long-term large-scale phosphorus fertilizer applications not only led to an accumulation of a large amount of phosphorus in the 0–20 cm soil layer but also increased phosphorus to varying degrees in the 20–40 cm soil layer. The inorganic phosphorus content of the protected-field soil was 0.29–1.24 g kg^−1^, accounting for 48.5–76.1% of total phosphorus; the organic phosphorus content was 0.24–0.78 g kg^−1^, accounting for 23.9–51.5% of total phosphorus; and the available phosphorus content was 0.09–0.57 g kg^−1^, accounting for 13.4–55.7% of total phosphorus, all of which, except for the percent organophosphorus, were significantly higher than that in farmland soil.

#### 3.1.2. Composition of Inorganic Phosphorus in Protected Fields

Table 4 and Table 5 show the values and proportions of inorganic phosphorus present as water-soluble and loose phosphorus (WSLC-P), aluminum phosphorus (Al-P), iron phosphorus (Fe-P), occluded phosphorus (O-P), and calcium phosphorus (Ca-P) in the 0–20 cm and 20–40 cm layers of the studied soils.

The tiny sample size might be the reason for the significant standard error among samples. In the 0-20 cm layer of the protected-field soils, Fe-P accounts for the majority of the inorganic phosphorus composition—followed by Al-P, Ca-P, and O-P—with WSLC-P representing the smallest proportion. The inorganic phosphorus composition of protected-field soils in the 20–40 cm layer differed slightly from that of the 0–20 cm soil layer, with Fe-P still dominating and O-P in relatively greater proportions.

### 3.2. Changes to Inorganic Phosphorus Content over Successive Planting Stubbles

#### 3.2.1. Changes to Water-Soluble and Loosely Combined Phosphorus (WSLC-P) Content over Successive Planting Stubbles

**Figure 1:**
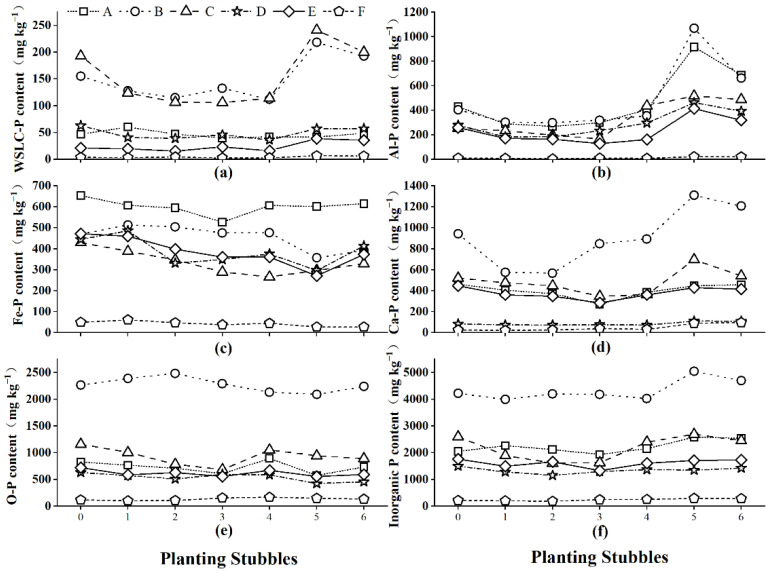
The changes of inorganic phosphorus sources in the studied soils over successive planting stubbles: (**a**) WSLC--P; (**b**) Al-P; (**c**) Fe-P; (**d**) Ca-P; (**e**) O-P; (**f**) total inorganic phosphorus.

Figure 1a shows that the WSLC-P content of farmland soil(F) changed slightly; the original soil content was 3.29 mg kg^−1^, which increased slightly after four planting stubbles to 5.85 mg kg^−1^ after six planting stubbles. In the protected-field soils, soil A decreased after the third cropping, then increased slightly because it was in a balanced state, while soils B, C, D, and E decreased over the first four planting stubbles but increased after the fourth and then decreased again after the fifth. This demonstrates that when WSLC-P content decreases to a certain extent due to absorption by plants, other forms of phosphorus will be rapidly converted into WSLC-P.

#### 3.2.2. Changes to Aluminum P (Al-P) Content over Successive Planting Stubbles

Figure 1b depicts a smooth change in Al-P content in dry land (F). Over the successive planting stubbles, the soil of protected-field soils (A, B, C, D, and E) had a fall-rise-fall trend. Soils A and B increased after the fourth and fifth planting stubble but showed an obvious downward trend after the sixth cropping, with the highest values reaching 915.61 mg kg^−1^ and 1068.32 mg kg^−1^, an increase of 53.29% and 62.24%, respectively. Soils C, D, and E had smaller increases, with the highest value reaching 517.21 mg kg^−1^, 461.53 mg kg^−1^ and 413.10 mg kg^−1^, an increase of 51.02%, 39.88%, and 37.77%, respectively. According to phosphorus content, protected-field soils were divided into a high-accumulation phosphorus group (A, B) and a low-accumulation phosphorus group (C, D, E). The high-accumulation phosphorus group maintained a steady phosphorus level for a longer time than the low-accumulation phosphorus group, and rates of increase and decrease were greater after the fourth cropping. This demonstrates that the high-phosphorus group provides a larger amount and longer-term availability of Al-P for plants and that the transformation rate of other forms of phosphorus into Al-P was also high.

#### 3.2.3. Changes to Iron P (Fe-P) Content over Successive Planting Stubbles

Figure 1c shows that the Fe-P content in the farmland soil (F) decreased overall, from 49.18 mg kg^−1^ to 27.04 mg kg^−1^ across the experiment. The protected-field soils (A, B, C, D, and E) decreased to varying degrees. The Fe-P of soils D and E increased after the fifth cropping, while that of soils A, B, and C remained unchanged, but the final content remained lower at the end of the experiment than at the beginning in all soils. The Fe-P content of the five protected-field soils decreased in 38.68 mg kg^−1^, 78.04 mg kg^−1^, 100.18 mg kg^−1^, 31.20 mg kg^−1^, and 97.75 mg kg^−1^ across the experiment. This shows that Fe-P in protected-field soils was absorbed by plants or transformed into other forms after multiple planting stubbles and could be used as an effective phosphorus source during plant growth. Furthermore, soil samples with high phosphorus content had low Fe-P conversion efficiency to other forms of phosphorus, whereas soil samples with low phosphorus content had high Fe-P conversion efficiency (except soil sample D, which was affected by texture).

#### 3.2.4. Changes to Calcium P (Ca-P) Content over Successive Planting Stubbles

As shown in Figure 1d, the Ca-P content of the farmland soil (F) did not change significantly before the fourth cropping and only showed a slow upward trend after the fifth and sixth cropping. The Ca-P content of protected-field soils A and E decreased after the first three crops were planted and increased after the last three plantings. The Ca-P content in protected-field soil C decreased after four planting stubbles and increased after the last two. In protected-field soil D, the Ca-P content changed in a similar way to that of the farmland soil. Other than soil sample B, where the Ca-P content changed dramatically, soil samples changed little. The reasons for the early declines are primarily plant growth and absorption, while the later increase is most likely due to transformation from other forms of phosphorus. This demonstrates that Ca-P is a phosphorus source for plants, and other forms of phosphorus can be converted into Ca-P when plant absorption of Ca-P decreases to a certain extent.

#### 3.2.5. Changes to Occluded P (O-P) Content over Successive Planting Stubbles

Figure 1e depicts a smooth change in O-P content in farmland soils (F). Apart from soil B, the O-P content of the protected-field soils decreased after the third cropping, then increased slightly before declining again through the remaining planting stubbles due to the aging trend of soil phosphate over increasing numbers of planting stubbles. Despite the fluctuating soil changes wrought by the planting and harvesting processes, the soil O-P content of protected-field soils remained relatively stable during the experiment, indicating that O-P content was less affected by other soil components, cannot be directly absorbed by plants, and cannot be easily converted into other forms. For plant growth, O-P can be used as a slow source of phosphorus because of its stability as a phosphorus pool.

#### 3.2.6. Changes to Total Inorganic Phosphorus Content over Successive Planting Stubbles

Figure 1f shows that the total content of inorganic phosphorus in the farmland soil (F) did not change significantly. The protected-field soils generally changed smoothly before the third cropping, with a slow upward trend after the fifth and sixth cropping. This demonstrates that though plants absorbed a large amount of inorganic phosphorus during the growth process, when inorganic phosphorus levels fell to a certain level, some organic phosphorus could be converted into inorganic phosphorus.

### 3.3. Changes to Available Phosphorus Content over Successive Planting Stubbles

**Figure 2:**
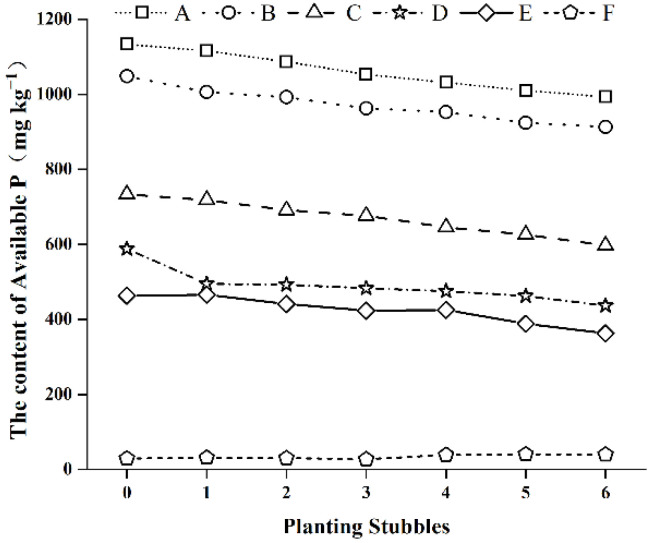
Changes in available P in the experimental soils over successive planting stubbles.

Figure 2 shows that the available phosphorus content in the farmland soil (F) did not change significantly with increasing numbers of planting stubbles, while that of the protected-field soils A, B, C, D, and E decreased, indicating that soil available phosphorus was absorbed by plants during growth and was the primary source of phosphorus for plant growth. The decrease in available phosphorus after a single planting stubble was lowest, at 100.24 mg kg^−1^, in soil sample E, and highest, at 151.24 mg kg^−1^, in soil sample D. The average decrease was 132.55 mg kg^−1^, indicating that plants can readily absorb the available phosphorus in soil. However, the overall average reduction from initial to final soil available phosphorus content was only 18.24%. The experiment found no significant differences in the yield of each protected-field soil after six planting stubbles, indicating that there was still more than enough phosphorus accumulated in the soil for plant growth.

### 3.4. Determination of the Critical Value of Phosphorus Deficiency and Establishment of a Prediction Model of Planting Stubbles

Data from the continuous-planting experiment were used to model the effect of soil available phosphorus on fresh weight of spinach. In the model, the soil available phosphorus content before a given planting is represented as *x*, and the fresh weight of spinach is represented as *y*. An exponential association model was formulated using Curve Expert 1.3. The resulting model is:

*y* = 60.344(1 − e^−0.023*x*^), R = 0.828 (1)

**Figure 3:**
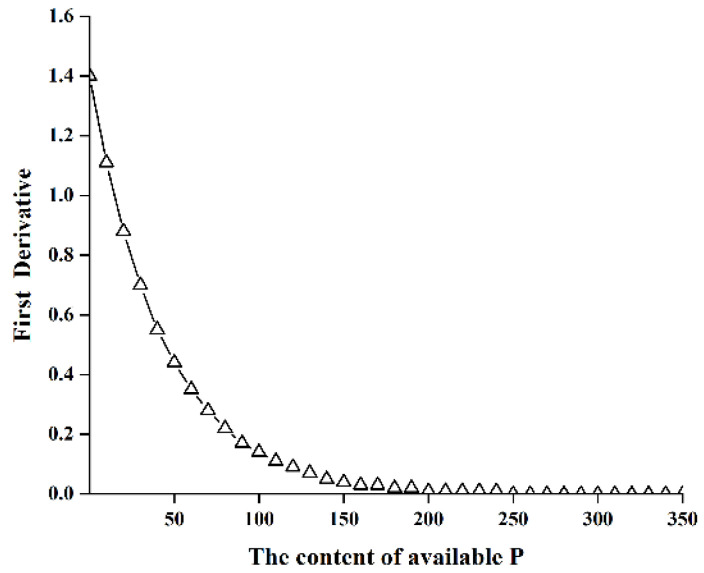
The first-order derivative of the equation modeling the change in the fresh weight of spinach with increasing soil available phosphorus.

Tests of the model proved it significantly fit the data, indicating it was available. The first-order derivative (Figure 3) was used to model the change in the fresh weight of spinach with increasing soil available phosphorus. When the first-order derivative approached zero, the rate of change of the fresh weight of spinach decreased. At this point, the fresh weight of spinach would not increase significantly even if the soil available phosphorus increased greatly. Based on this analysis, the critical value of phosphorus deficiency in spinach cultivated in the protected field is 200 mg kg^−1^.

The linear equation *y* = a + b*x* was fitted using soil available P content as the independent variable (*x*) and planting stubbles as the dependent variable (*y*) for each soil in the continuous-planting experiment to estimate the number of planting stubbles required to reduce the initial soil available phosphorus content to the critical value of spinach phosphorus deficiency. In other words, it would calculate the number of planting stubbles in which the demand for spinach phosphorus would be met. The results are shown in Table 6.

The following model was obtained using the data from Table 6 by fitting a linear model in which the initial, pre-planting available phosphorus in the protected-field soils predicts the estimated number of planting stubbles when the phosphorus requirements of spinach are met:

*y* = 4.0030 + 0.0295*x*, R = 0.99 (2)

in which *y* is the number of planting stubbles before the P requirements of spinach are no longer met, and *x* is the initial available P content in mg kg^−1^ where *x* > 200 mg kg^−1^).

This means you can predict the number of planting and harvesting stubbles in which the soil will meet the phosphorus demand of spinach if you know the initial soil available phosphorus (Bray-P) of protected-field soils cultivated on dark brown land, the soil texture type is similar, and spinach is grown in a manner similar to this experiment. This is a model for predicting phosphorus supply potential with broad application value.

## 4. Discussion

### 4.1. Soil Phosphorus Accumulation

Soil phosphorus nutrient status tends to be lower when the soil TP content is under 1 g kg^−1^ [25]. In our soil survey, protected-field soils at both sampled depths TP mean values are 1.76 g kg^−1^ and 1.00 g kg^−1^, which are greater than or equal to 1 g kg^−1^, which indicates that they were in a high nutrient state and provided ample phosphorus. Li et al. assessed the spatial distribution of soil P in orchards and farmland around the Danjiangkou Reservoir, China. The soil TP in orchards was 0.61 ± 0.23 g kg^−1^ [26], which was far below this study, because TP had strong spatial dependence, mainly influenced by soil properties (pH and SOM), precipitation, and topographic aspect.

According to [27], inorganic phosphorus (Pi) is the primary source of phosphorus in traditional farming systems. In this experiment, regardless of whether the soil was protected or open, Pi at both sampled depths (to 40 cm) accounted for the majority of soil total phosphorus (TP, Table 2 and Table 3), with the topsoil (0–20 cm) accumulating the majority This is consistent with previous research [28]. Phosphorus accumulation in soils is a major issue resulting from the widespread use of chemical fertilizers. The phosphorus content in the protected fields was nearly seven times that of the farmland with concentrations reaching up to 2.49 g kg^−1^, which is significantly higher than the average value of 0.086 g kg^−1^ reported in China [6]. Because vegetables have a short growth cycle and shallow root systems, they require more phosphorus in the topsoil [29]. As a result, the majority of the phosphorus in protected-field soils accumulates in the topsoil. According to the statistics, China’s greenhouse soil Olsen-P mean value was 179 mg kg^−1^ in 0~20cm soil depth [6], which was similar to the data in this study. According to [30], when the Olsen-P phosphorus content of the soil exceeds 25 mg kg^−1^, the soil’s phosphorus adsorption capacity becomes saturated, which results in a large amount of phosphorus leaching into groundwater or surface water, causing water eutrophication and agricultural non-point source pollution [31]. As a result, clarifying the dynamic changes and composition of soil phosphorus can help reduce the risk of pollution while also providing important information for future soil management.

### 4.2. Changes in Inorganic Phosphorus Content

Figure 1 shows that total inorganic phosphorus, Al-P, and Ca-P all increase slowly with the number of planting stubbles, which is consistent with previous research [32,33]. When soil available P is depleted, Al-P and Fe-P serve as potential P pools that can be converted into soluble P [15]. The Fe-P content showed a trend of first decreasing and then stable change, and the more acidic the soil, the greater the decrease of Fe-P. This is due to the acidic soil’s high microbial activity [34], which will adsorb and transform Fe-P to reduce residual fertilization-derived inositol hexakisphosphate (IHP) in the soil, exposing IHP to microbial degradation or moving it deeper into the soil [35]. In protected soil cultivation, pH decreases with the number of planting stubbles [32], which is favorable for the formation of Al-P and Ca-P [36]. In later planting, the content of Al-P and Ca-P were increased. In contrast, Liu et al. (2017) found that consumption of calcium-containing phosphate increased as soil pH decreased. Ca-P is primarily stored in a moderately available state (Ca_8_-P) in soils where phosphorus fertilizer has been applied for many years, so Ca_8_-P is an important potential phosphorus source for supplementing soil phosphorus [37]. In the latter period of this study, the change in O-P content was relatively stable and increased slowly, indicating that it was a slow-acting phosphorus source for plants.

### 4.3. Model Building

Soil phosphorus testing values should exceed the phosphorus deficit threshold for optimum crop yields, below which phosphorus becomes a limiting factor for crop yields [38]. The linear platform function method, the exponential decay function method, and the Mitscherlich function method are currently the three mainstream methods for building models [39]. Previous research has shown that using Bray-1 to fit the annual decline rate model of soil test P (STP) with an exponential decay function in areas without phosphate fertilizers is feasible [40]. The relative decay rate k = −0.023 and the constant a = 60.344 are both directly related to the initial Bray-P content in the model *y* = 60.344(1 − e^−0.023*x*^) predicting the effects of soil available phosphorus on the fresh weight of spinach [41]. The study by Hirte et al. showed that the determination of soil P threshold value is not only related to pedoclimatic conditions such as annual temperature or soil clay content, but soil pH also affects the threshold [42]. Among the various soil phosphorus tests available, the Bray-P method is most commonly used in soils with acidic to neutral pH [43]. The pH of the tested soils in this study was 5.47 on average. As a result, the Bray method was used in this paper to obtain a critical value of phosphorus deficiency of 200 mg kg^−1^ for spinach planted in protected fields through data analysis and model fitting. Olsen-P has a critical value of about 46 mg kg^−1^ on average in protected vegetable soils across China [6]. The critical value obtained in this paper is much higher because the Bray method measures soil available phosphorus as three times greater than the Olsen method [44].

The available phosphorus content of the soil and the predicted number of planting stubbles required to reduce soil phosphorus levels to the critical value were fitted with a linear model obtained: *y* = 4.0030 + 0.0295*x*, R = 0.99. Valkama et al. [45] (showed that the linear platform function and the Mitscherlich function have comparable goodness of fit and can provide better model fits than the quadratic function. Previous research has produced models predicting the number of planting stubbles based on the tested initial soil available phosphorus content, which, though like the prediction model obtained in this paper, are not applicable to all soil types. This study’s innovation is that the number of planting stubbles required to reach the critical value of phosphorus deficiency of vegetables was obtained for a type of soil with common soil texture types, and the data were simulated based on the initial soil available phosphorus content before pot planting and the predicted number of planting stubbles required to reach the critical value of vegetable. When these factors are combined, a specific prediction model can be obtained. In practice, the number of planting stubbles required to reach the phosphorus threshold value of spinach can only be determined by the soil Bray-P content.

## 5. Conclusions

In this study, soil WSLC-P, Al-P, Fe-P, and Ca-P first decreased and then increased over six planting stubbles of spinach in five protected-field soils. The overall change in O-P content was negligible. The total amount of soil inorganic phosphorus was stable across the early planting stubbles and rose slowly in the later stubbles. Soil available phosphorus, as the primary phosphorus source for plant growth, declined nearly linearly across the progressive planting stubbles. For growing spinach in a protected field, the threshold value of soil phosphorus deficiency is 200 mg kg^−1^. From this data, a prediction model of soil phosphorus supply potential was developed to describe the relationship between *x* (the soil available phosphorus) and *y* (the number of planting stubbles): *y* = 6.759 + 0.027*x*, R = 0.99. In practice, by just knowing the Bray-determined soil available phosphorus content, the number of planting stubbles required for available phosphorus to reach the critical value of phosphorus deficiency of spinach can be predicted using this model.

## Figures and Tables

**Table 1:** Basic properties of the tested continuous-planting experiment soils.

No.	Texture	pH	Available P	Hydrolyzed N	Available K	Total P	Total N	Total K	Organic Carbon
			mg kg^−1^			g kg^−1^			
A	sandy clay	5.25	1133.51	96.51	126.30	3.18	2.50	35.05	13.81
B	sandy clay	5.50	1048.16	143.44	138.95	5.55	2.01	37.22	18.49
C	sandy clay	5.54	733.36	86.35	136.96	3.25	2.31	34.97	11.42
D	clay	5.36	587.95	223.34	221.28	2.21	1.74	39.72	11.27
E	sandy clay	5.19	463.16	96.56	103.31	2.12	1.80	27.18	12.50
F	loamy clay	5.96	29.34	71.91	167.29	0.42	1.27	37.98	11.21

**Table 2:** Phosphorus composition of the top 20 cm of soil collected from protected fields.

No.	Type	Total P (TP) (g kg^−1^) ^1^	Inorganic P (Pi) (g kg^−1^) ^1^	Pi/TP % ^2^	Organophosphorus (Po) (g kg^−1^) ^1^	Po/TP % ^2^	Available P (Pav) (g kg^−1^) ^1^	Pav/TP % ^2^
1	Protected Field	2.45 ± 0.35	1.24 ± 0.49	50.5	1.21 ± 0.34	49.5	0.22 ± 0.04	9
2		2.03 ± 0.08	1.76 ± 0.35	86.9	0.27 ± 0.05	13.1	0.11 ± 0.02	5.6
3		2.49 ± 0.26	1.75 ± 0.17	70.4	0.74 ± 0.22	29.6	0.26 ± 0.01	10.6
4		0.80 ± 0.11	0.63 ± 0.20	78.5	0.17 ± 0.05	21.5	0.13 ± 0.05	16.7
5		0.80 ± 0.06	0.64 ± 0.12	80	0.16 ± 0.06	20	0.04 ± 0.01	5.1
6		1.28 ± 0.18	0.92 ± 0.13	71.7	0.36 ± 0.03	28.3	0.18 ± 0.02	14.3
7		2.43 ± 0.06	1.61 ± 0.29	66.1	0.82 ± 0.16	33.9	0.53 ± 0.12	21.8
8		1.77 ± 0.15	1.37 ± 0.46	77.5	0.40 ± 0.11	22.5	0.22 ± 0.02	12.2
9	Farmland	0.36 ± 0.02	0.27 ± 0.08	75.1	0.09 ± 0.02	24.9	0.03 ± 0.01	9.6

^1^ The values in the table represent the mean ± standard error; ^2^ each form’s phosphorus content/total phosphorus × 100.

**Table 3:** Phosphorus composition of the 20–40 cm layer of soils collected from protected fields.

No.	Type	Total P (TP) (g kg^−1^) ^1^	Inorganic P (Pi) (g kg^−1^) ^1^	Pi/TP % ^2^	Organophosphorus (Po) (g kg^−1^) ^1^	Po/TP % ^2^	Available P (Pav) (g kg^−1^) ^1^	Pav/TP % ^2^
1	Protected Field	0.70 ± 0.31	0.44 ± 0.15	62.4	0.26 ± 0.08	37.6	0.23 ± 0.02	32.8
2		0.61 ± 0.18	0.29 ± 0.14	48.5	0.32 ± 0.09	51.5	0.15 ± 0.02	24.5
3		1.02 ± 0.64	0.77 ± 0.20	75.4	0.25 ± 0.03	24.6	0.57 ± 0.04	55.7
4		0.74 ± 0.25	0.48 ± 0.15	65.5	0.26 ± 0.03	34.5	0.16 ± 0.03	21.3
5		0.65 ± 0.22	0.36 ± 0.19	54.6	0.29 ± 0.04	45.4	0.09 ± 0.02	13.4
6		1.03 ± 0.45	0.79 ± 0.21	76.1	0.24 ± 0.02	23.9	0.23 ± 0.04	22
7		2.02 ± 0.67	1.24 ± 0.32	61.5	0.78 ± 0.14	38.5	0.48 ± 0.07	23.9
8		1.25 ± 0.43	0.91 ± 0.41	72.5	0.34 ± 0.02	27.5	0.21 ± 0.09	17
9	Farmland	0.34 ± 0.24	0.16 ± 0.10	46.2	0.18 ± 0.09	53.8	0.04 ± 0.01	11.1

^1^ The values in the table represent the mean ± standard error; ^2^ each form’s phosphorus content/total phosphorus × 100.

**Table 4:** The composition of inorganic phosphorus in the top 20 cm of soils collected from protected fields ^1^.

No.	Type	WSLC-P		Al-P		Fe-P		O-P		Ca-P	
		mg kg^−1^	% ^2^	mg kg^−1^	%^2^	mg kg^−1^	% ^2^	mg kg^−1^	% ^2^	mg kg^−1^	% ^2^
1	Protected Field	173.02 ± 7.56	14.0	260.27 ± 8.93	21.0	264.81 ± 1.26	21.4	179.51 ± 9.88	14.5	361.22 ± 4.02	29.2
2		227.84 ± 8.66	12.9	427.81 ± 2.54	24.3	368.85 ± 7.62	20.9	171.83 ± 7.87	9.7	567.84 ± 3.39	32.2
3		42.96 ± 2.83	2.5	534.94 ± 4.40	30.6	362.63 ± 4.46	20.7	450.90 ± 2.65	25.8	357.87 ± 6.34	20.5
4		25.00 ± 2.98	4.0	99.64 ± 1.39	15.9	247.54 ± 1.61	39.6	142.78 ± 9.09	22.8	110.06 ± 3.28	17.6
5		20.62 ± 3.67	3.2	161.43 ± 7.41	25.1	356.12 ± 3.87	55.5	56.19 ± 2.20	8.8	47.69 ± 2.36	7.4
6		72.04 ± 8.93	7.9	256.32 ± 3.85	27.9	277.49 ± 4.43	30.2	50.75 ± 1.57	5.5	260.83 ± 9.48	28.4
7		40.13 ± 4.33	2.5	420.18 ± 5.33	26.1	650.46 ± 5.01	40.5	182.97 ± 9.83	11.4	313.07 ± 1.21	19.5
8		35.76 ± 3.47	2.6	161.34 ± 5.45	11.8	655.42 ± 4.74	47.9	177.25 ± 9.19	13.0	338.93 ± 6.12	24.8
9	Farmland	34.10 ± 2.44	12.6	45.99 ± 8.09	17.0	33.05 ± 3.14	12.2	62.66 ± 2.64	23.2	94.85 ± 3.54	35.0

^1^ The values in the table represent the mean ± standard error; ^2^ each form’s phosphorus content/total inorganic phosphorus × 100.

**Table 5:** The composition of inorganic phosphorus in the 20–40 cm layer of soils collected from protected fields ^1^.

No.	Type	WSLC-P		Al-P		Fe-P		O-P		Ca-P	
		mg kg^−1^	%^2^	mg kg^−1^	% ^2^	mg kg^−1^	% ^2^	mg kg^−1^	% ^2^	mg kg^−1^	% ^2^
1	Protected Field	13.57 ± 0.66	3.1	97.55 ± 10.1	22.3	108.05 ± 22.69	24.7	110.08 ± 17.38	25.2	108.23 ± 19.41	24.7
2		17.00 ± 4.03	5.8	32.58 ± 2.0	11.1	95.06 ± 15.19	32.4	113.27 ± 10.45	38.6	35.91 ± 1.77	12.2
3		21.23 ± 1.55	2.7	227.92 ± 29.7	29.5	311.77 ± 6.08	40.3	180.31 ± 4.58	23.3	31.57 ± 2.77	4.1
4		19.17 ± 1.41	4.0	31.48 ± 10.8	6.5	123.06 ± 11.77	25.6	227.46 ± 9.62	47.2	80.33 ± 3.66	16.7
5		6.06 ± 0.92	1.7	29.35 ± 3.7	8.2	126.13 ± 21.40	35.4	151.54 ± 5.36	42.5	43.53 ± 1.55	12.2
6		20.69 ± 0.93	2.6	81.20 ± 7.4	10.3	283.61 ± 51.50	36.1	243.37 ± 22.76	31.0	156.96 ± 23.43	20.0
7		18.27 ± 1.47	1.5	281.09 ± 12.0	22.6	471.89 ± 58.59	38.0	305.52 ± 34.92	24.6	164.83 ± 21.87	13.3
8		21.74 ± 2.00	2.4	204.36 ± 10.6	22.5	460.36 ± 46.47	50.6	131.11 ± 11.38	14.4	91.98 ± 14.51	10.1
9	Farmland	6.43 ± 1.56	4.1	13.37 ± 8.5	8.4	33.93 ± 3.69	21.4	59.57 ± 11.49	37.6	45.29 ± 1.67	28.6

^1^ The values in the table represent the mean ±standard error; ^2^ each form’s phosphorus content/total inorganic phosphorus × 100.

**Table 6:** Fitted model between planting stubbles and content of available phosphorus in a protected field and the number of planting stubbles required for the phosphorus needs of spinach to be satisfied.

No.	Fitted Model	R	Number of Planting Stubbles	Initial Available P (mg kg^−1^)
A	*y* = 44.670 − 0.038*x*	0.996	37	1133.51
B	*y* = 44.746 − 0.0420*x*	0.986	36	1048.16
C	*y* = 34.250 − 0.045*x*	0.996	25	733.36
D	*y* = 118.159 − 18.463 × ln(*x*)	0.841	20	587.95
E	*y* = 34.087 − 0.0711*x*	0.957	19	463.16

## Data Availability

The authors declare that all relevant data supporting the findings of the study are available within the manuscript.
